# *catena*-Poly[[(liriodenine-κ^2^ *N*,*O*)lead(II)]-di-μ-chlorido]

**DOI:** 10.1107/S1600536809051381

**Published:** 2009-12-09

**Authors:** Yan-Tao Qin, Lu Tao, Tian-Jing He, Yan-Cheng Liu, Zhen-Feng Chen

**Affiliations:** a Key Laboratory for the Chemistry and Molecular Engineering of Medicinal Resources (Ministry of Education of China), School of Chemistry & Chemical Engineering, Guangxi Normal University, Guilin 541004, People’s Republic of China

## Abstract

The title compound, [PbCl_2_(C_17_H_9_NO_3_)]_*n*_, was synthesized by the hydro­thermal reaction of PbCl_2_ and liriodenine. The lead(II) atom has a distorted octa­hedral environment made up of the O and N atoms of the liriodenine ligand [Pb—O 2.666 (4) Å, Pb—N 2.587 (5) Å, O—Pb—N 61.78 (14)°] and four bridging chloro ligands, which link the complex mol­ecules into infinite chains along the *a* axis. Both crystallographically independent chloro-bridges are asymmetric, so that the Pb atom participates in two short [2.6872 (18) and 2.7952 (18) Å] and two noticeably longer Pb—Cl bonds [2.9626 (18) and 3.031 (2) Å].

## Related literature

For liriodenine metal complexes, see: Chen *et al.* (2009 ▶). For the structure of a similar lead(II) coordination polymer, see: Engelhardt *et al.* (1987 ▶).
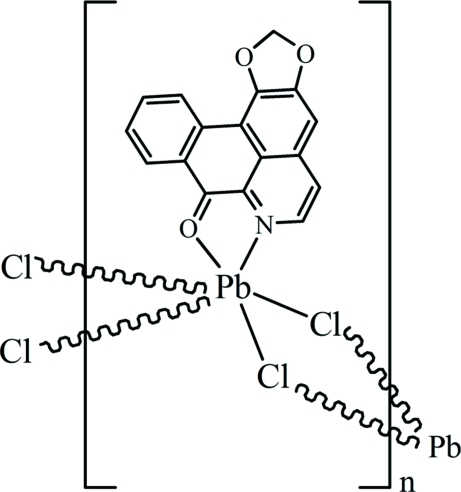


## Experimental

### 

#### Crystal data

[PbCl_2_(C_17_H_9_NO_3_)]
*M* *_r_* = 553.34
Triclinic, 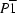

*a* = 7.2280 (18) Å
*b* = 10.332 (3) Å
*c* = 11.307 (3) Å
α = 104.481 (6)°
β = 100.479 (4)°
γ = 99.686 (4)°
*V* = 783.4 (3) Å^3^
*Z* = 2
Mo *K*α radiation
μ = 11.13 mm^−1^
*T* = 293 K
0.35 × 0.20 × 0.15 mm

#### Data collection

Rigaku Mercury CCD diffractometer
Absorption correction: multi-scan (*REQAB*; Jacobson, 1998 ▶) *T*_min_ = 0.077, *T*_max_ = 0.188
7685 measured reflections
2847 independent reflections
2545 reflections with *I* > 2σ(*I*)
*R*_int_ = 0.040

#### Refinement

*R*[*F* ^2^ > 2σ(*F* ^2^)] = 0.033
*wR*(*F* ^2^) = 0.065
*S* = 1.05
2847 reflections
218 parameters
H-atom parameters constrained
Δρ_max_ = 1.19 e Å^−3^
Δρ_min_ = −1.28 e Å^−3^

### 

Data collection: *CrystalClear* (Rigaku, 1999 ▶); cell refinement: *CrystalClear*; data reduction: *CrystalStructure* (Rigaku/MSC & Rigaku, 2000 ▶); program(s) used to solve structure: *SHELXS97* (Sheldrick, 2008 ▶); program(s) used to refine structure: *SHELXL97* (Sheldrick, 2008 ▶); molecular graphics: *SHELXTL* (Sheldrick, 2008 ▶); software used to prepare material for publication: *SHELXTL*.

## Supplementary Material

Crystal structure: contains datablocks I, New_Global_Publ_Block. DOI: 10.1107/S1600536809051381/ya2112sup1.cif

Structure factors: contains datablocks I. DOI: 10.1107/S1600536809051381/ya2112Isup2.hkl

Additional supplementary materials:  crystallographic information; 3D view; checkCIF report

## Figures and Tables

**Fig. 1.**
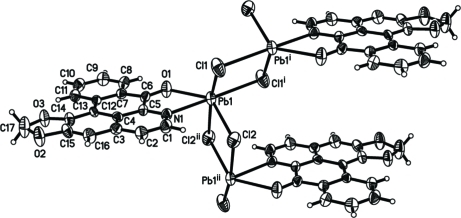
Molecular structure of the title compound, showing the atom-labelling scheme; displacement ellipsoids are drawn at the 50% probability level. Symmetry transformations (i): -x + 1, -y + 1, -z + 1; (ii): -x,-y + 1,-z + 1.

## supplementary crystallographic information

### Comment

Liriodenine, 8*H*-[1,3]benzodioxolo[6,5,4-de]benzo[*g*]quinolin-8-one, is an oxo-aporphine alkaloid, which was isolated from the *Z*. nitidum (TCM) spiders found in China (Chen *et al.*, 2009). With its N and carbonyl O donor atoms, liriodenine can serve as bidentate chelate ligand in metal complex. In our previous work, the synthesis, crystal structures and anticancer activity of platinum(II) and ruthenium(II) complexes of liriodenine were reported (Chen *et al.*, 2009). In order to extend our knowledge on liriodenine coordination chemistry we turned to the main-group metals and report herein the the first structure of lead(II) complex with liriodenine.

As shown in Fig.1, similarly to what was observed in the structure of *catena*-[*cis*-bis(µ_2_-chloro)-(3-methylpyridine-N)]lead(II) (Engelhardt *et al.*, 1987), the Pb1 atom in the title compound is six-coordinated by the O1 and N1 atoms of the liriodenine ligand [Pb1—O1 2.666 (4) Å, Pb1—N1 2.587 (5) Å] and four µ_2_-chloro-atoms which link the complex molecules into infinite chains running along the *a* axis. The chloro-bridges show noticeable asymmetry with the Pb1—Cl1 [2.7952 (18) Å] and Pb1—Cl2 [2.6872 (18) Å] bonds being significantly shorter than Pb1—Cl1^ii^ [3.031 (2) Å], and Pb1—Cl2^i^ [2.9626 (18) Å] (see Fig. 1). The octahedral coordination of the Pb1 atom shows considerable distortion due to the presence of the chelate ligand [angle O1—Pb1—N1 is equal to 61.78 (14)°] and the asymmetry of the chloro-bridges, *e.g.* the N1—Pb1—Cl2 and O1—Pb1—Cl2 angles are 84.80 (11)° and 136.98 (11)°, respectively. The overall geometry of the complex compares quite well with that of *catena*-(*cis*-bis((µ_2_-chloro)-(3-methylpyridine-N)) lead(II) (Engelhardt *et al.* 1987), and the geometric parameters of liriodenine are close to those reported previously (Chen *et al.*, 2009).

### Experimental

PbCl_2_ (0.8 mmol, 0.222 g) and liriodenine (0.8 mmol, 0.220 g) were thoroughly mixed in a mortar with a pestle, and placed in a thick-walled Pyrex tube (*ca* 20 cm long). After addition of EtOH (0.6 ml) and H_2_O (0.3 ml), the tube was frozen with liquid nitrogen, evacuated under vacuum and sealed with a torch. The tube was heated at 110°C for 2 days and then slowly cooled down to room temperature; brown-red block crystals were obtained. Yield: 40%.

### Refinement

The H atoms bonded to C atoms were positioned geometrically (C—H 0.93 Å for aromatic and 0.97 Å for aliphatic qroups). and included in the refinement in riding model approximation with *U*_iso_(H) = 1.2*U*_eq_(C). The highest peak of 1.19 e Å^-3^ is located at 1.65 Å from O3; the deepest hole of -1.28 is found at a distance of 0.94 Å from Pb1.

### Crystal data

**Table:**

[PbCl_2_(C_17_H_9_NO_3_)]	*Z* = 2
*M**_r_* = 553.34	*F*(000) = 516
Triclinic, *P*1	*D*_x_ = 2.346 Mg m^−^^3^
Hall symbol: -P 1	Mo *K*α radiation, λ = 0.71070 Å
*a* = 7.2280 (18) Å	Cell parameters from 3068 reflections
*b* = 10.332 (3) Å	θ = 3.1–25.3°
*c* = 11.307 (3) Å	µ = 11.13 mm^−^^1^
α = 104.481 (6)°	*T* = 293 K
β = 100.479 (4)°	Block, brown-red
γ = 99.686 (4)°	0.35 × 0.20 × 0.15 mm
*V* = 783.4 (3) Å^3^	

### Data collection

**Table:**

Rigaku Mercury CCD diffractometer	2847 independent reflections
Radiation source: fine-focus sealed tube	2545 reflections with *I* > 2σ(*I*)
graphite	*R*_int_ = 0.040
Detector resolution: 7.31 pixels mm^-1^	θ_max_ = 25.3°, θ_min_ = 3.1°
ω scans	*h* = −8→8
Absorption correction: multi-scan (REQAB; Jacobson, 1998)	*k* = −12→12
*T*_min_ = 0.077, *T*_max_ = 0.188	*l* = −13→11
7685 measured reflections	

### Refinement

**Table:**

Refinement on *F*^2^	Primary atom site location: structure-invariant direct methods
Least-squares matrix: full	Secondary atom site location: difference Fourier map
*R*[*F*^2^ > 2σ(*F*^2^)] = 0.033	Hydrogen site location: inferred from neighbouring sites
*wR*(*F*^2^) = 0.065	H-atom parameters constrained
*S* = 1.05	*w* = 1/[σ^2^(*F*_o_^2^) + (0.0269*P*)^2^] where *P* = (*F*_o_^2^ + 2*F*_c_^2^)/3
2847 reflections	(Δ/σ)_max_ < 0.001
218 parameters	Δρ_max_ = 1.19 e Å^−^^3^
0 restraints	Δρ_min_ = −1.28 e Å^−^^3^

### Special details

**Table:**

Geometry. All e.s.d.'s (except the e.s.d. in the dihedral angle between two l.s. planes) are estimated using the full covariance matrix. The cell e.s.d.'s are taken into account individually in the estimation of e.s.d.'s in distances, angles and torsion angles; correlations between e.s.d.'s in cell parameters are only used when they are defined by crystal symmetry. An approximate (isotropic) treatment of cell e.s.d.'s is used for estimating e.s.d.'s involving l.s. planes.
Refinement. Refinement of *F*^2^ against ALL reflections. The weighted *R*-factor *wR* and goodness of fit *S* are based on *F*^2^, conventional *R*-factors *R* are based on *F*, with *F* set to zero for negative *F*^2^. The threshold expression of *F*^2^ > σ(*F*^2^) is used only for calculating *R*-factors(gt) *etc*. and is not relevant to the choice of reflections for refinement. *R*-factors based on *F*^2^ are statistically about twice as large as those based on *F*, and *R*- factors based on ALL data will be even larger.

### Fractional atomic coordinates and isotropic or equivalent isotropic displacement parameters (Å^2^)

**Table:**

	*x*	*y*	*z*	*U*_iso_*/*U*_eq_	
Pb1	0.19801 (4)	0.42620 (2)	0.40684 (2)	0.03726 (11)	
Cl1	−0.1055 (3)	0.29827 (18)	0.4893 (2)	0.0629 (6)	
Cl2	0.4274 (3)	0.44005 (18)	0.62730 (15)	0.0502 (5)	
O1	0.1822 (7)	0.3210 (4)	0.1636 (4)	0.0497 (12)	
O2	0.3150 (8)	−0.4078 (5)	0.0294 (4)	0.0578 (14)	
O3	0.2905 (7)	−0.2806 (4)	−0.1103 (4)	0.0480 (12)	
N1	0.2587 (7)	0.1823 (5)	0.3329 (4)	0.0326 (12)	
C1	0.2820 (9)	0.1075 (7)	0.4135 (6)	0.0398 (16)	
H1	0.2900	0.1488	0.4979	0.048*	
C2	0.2945 (9)	−0.0270 (7)	0.3773 (6)	0.0395 (15)	
H2	0.3066	−0.0751	0.4366	0.047*	
C3	0.2895 (8)	−0.0921 (6)	0.2529 (5)	0.0313 (14)	
C4	0.2695 (8)	−0.0126 (6)	0.1654 (5)	0.0273 (13)	
C5	0.2517 (8)	0.1231 (6)	0.2115 (5)	0.0276 (13)	
C6	0.2153 (8)	0.2068 (6)	0.1253 (5)	0.0302 (14)	
C7	0.2194 (8)	0.1492 (6)	−0.0059 (5)	0.0303 (14)	
C8	0.1938 (9)	0.2315 (6)	−0.0863 (6)	0.0365 (15)	
H8	0.1783	0.3203	−0.0549	0.044*	
C9	0.1914 (9)	0.1812 (7)	−0.2120 (6)	0.0420 (16)	
H9	0.1775	0.2360	−0.2655	0.050*	
C10	0.2102 (9)	0.0463 (7)	−0.2573 (6)	0.0447 (17)	
H10	0.2059	0.0111	−0.3421	0.054*	
C11	0.2351 (9)	−0.0361 (6)	−0.1792 (5)	0.0349 (15)	
H11	0.2488	−0.1250	−0.2117	0.042*	
C12	0.2399 (7)	0.0135 (6)	−0.0515 (5)	0.0252 (13)	
C13	0.2642 (8)	−0.0694 (6)	0.0357 (5)	0.0259 (13)	
C14	0.2808 (9)	−0.2026 (6)	0.0034 (6)	0.0360 (15)	
C15	0.2995 (9)	−0.2795 (6)	0.0903 (6)	0.0374 (15)	
C16	0.3033 (9)	−0.2300 (6)	0.2118 (6)	0.0401 (16)	
H16	0.3144	−0.2837	0.2666	0.048*	
C17	0.2998 (14)	−0.4135 (7)	−0.0990 (7)	0.066 (2)	
H17A	0.1845	−0.4802	−0.1511	0.079*	
H17B	0.4111	−0.4405	−0.1261	0.079*	

### Atomic displacement parameters (Å^2^)

**Table:**

	*U*^11^	*U*^22^	*U*^33^	*U*^12^	*U*^13^	*U*^23^
Pb1	0.04943 (18)	0.03102 (16)	0.03286 (16)	0.01243 (12)	0.01518 (11)	0.00575 (10)
Cl1	0.0760 (14)	0.0351 (10)	0.0866 (14)	0.0132 (9)	0.0457 (12)	0.0144 (9)
Cl2	0.0624 (12)	0.0544 (11)	0.0345 (9)	0.0071 (9)	0.0160 (8)	0.0146 (8)
O1	0.086 (4)	0.034 (3)	0.032 (3)	0.023 (3)	0.013 (2)	0.008 (2)
O2	0.101 (4)	0.032 (3)	0.048 (3)	0.027 (3)	0.027 (3)	0.011 (2)
O3	0.080 (4)	0.032 (3)	0.035 (3)	0.022 (2)	0.020 (2)	0.005 (2)
N1	0.040 (3)	0.033 (3)	0.024 (3)	0.011 (2)	0.006 (2)	0.008 (2)
C1	0.053 (4)	0.044 (4)	0.024 (3)	0.016 (3)	0.007 (3)	0.009 (3)
C2	0.052 (4)	0.038 (4)	0.034 (4)	0.017 (3)	0.011 (3)	0.015 (3)
C3	0.033 (3)	0.032 (4)	0.030 (3)	0.009 (3)	0.006 (3)	0.012 (3)
C4	0.022 (3)	0.028 (3)	0.032 (3)	0.006 (3)	0.009 (2)	0.008 (3)
C5	0.031 (3)	0.023 (3)	0.027 (3)	0.006 (3)	0.007 (2)	0.004 (2)
C6	0.032 (3)	0.023 (3)	0.029 (3)	0.002 (3)	0.004 (3)	0.002 (3)
C7	0.028 (3)	0.031 (3)	0.030 (3)	0.004 (3)	0.008 (3)	0.006 (3)
C8	0.047 (4)	0.030 (4)	0.031 (3)	0.003 (3)	0.009 (3)	0.010 (3)
C9	0.055 (4)	0.037 (4)	0.040 (4)	0.010 (3)	0.014 (3)	0.019 (3)
C10	0.049 (4)	0.053 (5)	0.028 (3)	0.006 (4)	0.014 (3)	0.006 (3)
C11	0.039 (4)	0.034 (4)	0.031 (3)	0.006 (3)	0.012 (3)	0.007 (3)
C12	0.023 (3)	0.024 (3)	0.028 (3)	0.004 (2)	0.008 (2)	0.004 (2)
C13	0.025 (3)	0.023 (3)	0.030 (3)	0.009 (2)	0.010 (2)	0.003 (2)
C14	0.043 (4)	0.033 (4)	0.033 (4)	0.011 (3)	0.017 (3)	0.003 (3)
C15	0.046 (4)	0.024 (3)	0.048 (4)	0.014 (3)	0.018 (3)	0.012 (3)
C16	0.054 (4)	0.031 (4)	0.044 (4)	0.013 (3)	0.018 (3)	0.018 (3)
C17	0.116 (7)	0.034 (4)	0.046 (5)	0.021 (4)	0.024 (5)	0.003 (3)

### Geometric parameters (Å, °)

**Table:**

Pb1—N1	2.587 (5)	C4—C5	1.405 (8)
Pb1—O1	2.666 (4)	C4—C13	1.428 (7)
Pb1—Cl2	2.6872 (18)	C5—C6	1.474 (8)
Pb1—Cl1	2.7952 (18)	C6—C7	1.462 (8)
Pb1—Cl2^i^	2.9626 (18)	C7—C8	1.401 (8)
Pb1—Cl1^ii^	3.031 (2)	C7—C12	1.411 (8)
Cl1—Pb1^ii^	3.031 (2)	C8—C9	1.382 (8)
Cl2—Pb1^i^	2.9626 (18)	C8—H8	0.9300
O1—C6	1.230 (7)	C9—C10	1.399 (9)
O2—C15	1.366 (7)	C9—H9	0.9300
O2—C17	1.422 (8)	C10—C11	1.380 (9)
O3—C14	1.356 (7)	C10—H10	0.9300
O3—C17	1.422 (8)	C11—C12	1.397 (8)
N1—C1	1.340 (7)	C11—H11	0.9300
N1—C5	1.344 (7)	C12—C13	1.465 (8)
C1—C2	1.370 (9)	C13—C14	1.365 (8)
C1—H1	0.9300	C14—C15	1.412 (8)
C2—C3	1.390 (8)	C15—C16	1.334 (8)
C2—H2	0.9300	C16—H16	0.9300
C3—C16	1.411 (8)	C17—H17A	0.9700
C3—C4	1.438 (8)	C17—H17B	0.9700
			
N1—Pb1—O1	61.78 (14)	O1—C6—C5	120.5 (5)
N1—Pb1—Cl2	84.80 (11)	C7—C6—C5	117.8 (5)
O1—Pb1—Cl2	136.98 (11)	C8—C7—C12	121.0 (5)
N1—Pb1—Cl1	84.76 (11)	C8—C7—C6	117.6 (5)
O1—Pb1—Cl1	114.05 (11)	C12—C7—C6	121.3 (5)
Cl2—Pb1—Cl1	86.77 (6)	C9—C8—C7	120.2 (6)
N1—Pb1—Cl2^i^	93.28 (11)	C9—C8—H8	119.9
O1—Pb1—Cl2^i^	75.61 (11)	C7—C8—H8	119.9
Cl2—Pb1—Cl2^i^	80.49 (5)	C8—C9—C10	118.7 (6)
Cl1—Pb1—Cl2^i^	167.23 (6)	C8—C9—H9	120.6
N1—Pb1—Cl1^ii^	174.35 (11)	C10—C9—H9	120.6
O1—Pb1—Cl1^ii^	122.56 (10)	C11—C10—C9	121.7 (6)
Cl2—Pb1—Cl1^ii^	92.96 (6)	C11—C10—H10	119.2
Cl1—Pb1—Cl1^ii^	89.95 (5)	C9—C10—H10	119.2
Cl2^i^—Pb1—Cl1^ii^	91.45 (5)	C10—C11—C12	120.4 (6)
Pb1—Cl1—Pb1^ii^	90.05 (5)	C10—C11—H11	119.8
Pb1—Cl2—Pb1^i^	99.51 (5)	C12—C11—H11	119.8
C6—O1—Pb1	120.2 (4)	C11—C12—C7	118.0 (5)
C15—O2—C17	107.0 (5)	C11—C12—C13	122.9 (5)
C14—O3—C17	107.2 (5)	C7—C12—C13	119.1 (5)
C1—N1—C5	118.2 (5)	C14—C13—C4	114.7 (5)
C1—N1—Pb1	120.4 (4)	C14—C13—C12	125.1 (5)
C5—N1—Pb1	121.2 (4)	C4—C13—C12	120.3 (5)
N1—C1—C2	123.1 (5)	O3—C14—C13	127.8 (6)
N1—C1—H1	118.5	O3—C14—C15	109.0 (5)
C2—C1—H1	118.5	C13—C14—C15	123.1 (5)
C1—C2—C3	120.7 (6)	C16—C15—O2	127.3 (6)
C1—C2—H2	119.7	C16—C15—C14	123.8 (6)
C3—C2—H2	119.7	O2—C15—C14	108.8 (5)
C2—C3—C16	122.5 (5)	C15—C16—C3	116.5 (6)
C2—C3—C4	117.1 (5)	C15—C16—H16	121.8
C16—C3—C4	120.4 (5)	C3—C16—H16	121.8
C5—C4—C13	120.8 (5)	O2—C17—O3	107.6 (5)
C5—C4—C3	117.6 (5)	O2—C17—H17A	110.2
C13—C4—C3	121.6 (5)	O3—C17—H17A	110.2
N1—C5—C4	123.2 (5)	O2—C17—H17B	110.2
N1—C5—C6	116.2 (5)	O3—C17—H17B	110.2
C4—C5—C6	120.5 (5)	H17A—C17—H17B	108.5
O1—C6—C7	121.7 (5)		
			
N1—Pb1—Cl1—Pb1^ii^	178.04 (11)	N1—C5—C6—C7	176.0 (5)
O1—Pb1—Cl1—Pb1^ii^	−126.08 (11)	C4—C5—C6—C7	−6.5 (8)
Cl2—Pb1—Cl1—Pb1^ii^	92.96 (6)	O1—C6—C7—C8	3.1 (9)
Cl2^i^—Pb1—Cl1—Pb1^ii^	96.4 (2)	C5—C6—C7—C8	−177.1 (5)
Cl1^ii^—Pb1—Cl1—Pb1^ii^	0.0	O1—C6—C7—C12	−174.1 (5)
N1—Pb1—Cl2—Pb1^i^	94.22 (11)	C5—C6—C7—C12	5.7 (8)
O1—Pb1—Cl2—Pb1^i^	56.71 (16)	C12—C7—C8—C9	−1.1 (9)
Cl1—Pb1—Cl2—Pb1^i^	179.24 (6)	C6—C7—C8—C9	−178.3 (6)
Cl2^i^—Pb1—Cl2—Pb1^i^	0.0	C7—C8—C9—C10	1.6 (9)
Cl1^ii^—Pb1—Cl2—Pb1^i^	−90.98 (6)	C8—C9—C10—C11	−1.4 (10)
N1—Pb1—O1—C6	−1.9 (4)	C9—C10—C11—C12	0.8 (10)
Cl2—Pb1—O1—C6	41.5 (5)	C10—C11—C12—C7	−0.3 (8)
Cl1—Pb1—O1—C6	−71.3 (5)	C10—C11—C12—C13	179.4 (6)
Cl2^i^—Pb1—O1—C6	99.9 (5)	C8—C7—C12—C11	0.4 (8)
Cl1^ii^—Pb1—O1—C6	−177.8 (4)	C6—C7—C12—C11	177.6 (5)
O1—Pb1—N1—C1	−174.8 (5)	C8—C7—C12—C13	−179.3 (5)
Cl2—Pb1—N1—C1	33.3 (4)	C6—C7—C12—C13	−2.2 (8)
Cl1—Pb1—N1—C1	−53.9 (4)	C5—C4—C13—C14	−179.2 (5)
Cl2^i^—Pb1—N1—C1	113.5 (4)	C3—C4—C13—C14	−0.5 (8)
O1—Pb1—N1—C5	−0.4 (4)	C5—C4—C13—C12	0.0 (8)
Cl2—Pb1—N1—C5	−152.2 (4)	C3—C4—C13—C12	178.8 (5)
Cl1—Pb1—N1—C5	120.5 (4)	C11—C12—C13—C14	−1.3 (9)
Cl2^i^—Pb1—N1—C5	−72.1 (4)	C7—C12—C13—C14	178.4 (5)
C5—N1—C1—C2	−1.5 (9)	C11—C12—C13—C4	179.5 (5)
Pb1—N1—C1—C2	173.0 (5)	C7—C12—C13—C4	−0.8 (8)
N1—C1—C2—C3	2.0 (10)	C17—O3—C14—C13	−177.9 (7)
C1—C2—C3—C16	179.6 (6)	C17—O3—C14—C15	4.7 (7)
C1—C2—C3—C4	−0.4 (9)	C4—C13—C14—O3	−176.3 (6)
C2—C3—C4—C5	−1.6 (8)	C12—C13—C14—O3	4.5 (10)
C16—C3—C4—C5	178.4 (5)	C4—C13—C14—C15	0.8 (9)
C2—C3—C4—C13	179.6 (5)	C12—C13—C14—C15	−178.5 (6)
C16—C3—C4—C13	−0.4 (8)	C17—O2—C15—C16	179.0 (7)
C1—N1—C5—C4	−0.7 (8)	C17—O2—C15—C14	−1.8 (8)
Pb1—N1—C5—C4	−175.2 (4)	O3—C14—C15—C16	177.3 (6)
C1—N1—C5—C6	176.8 (5)	C13—C14—C15—C16	−0.2 (10)
Pb1—N1—C5—C6	2.2 (7)	O3—C14—C15—O2	−1.8 (7)
C13—C4—C5—N1	−179.0 (5)	C13—C14—C15—O2	−179.4 (6)
C3—C4—C5—N1	2.2 (8)	O2—C15—C16—C3	178.3 (6)
C13—C4—C5—C6	3.7 (8)	C14—C15—C16—C3	−0.7 (10)
C3—C4—C5—C6	−175.1 (5)	C2—C3—C16—C15	−179.0 (6)
Pb1—O1—C6—C7	−176.2 (4)	C4—C3—C16—C15	0.9 (9)
Pb1—O1—C6—C5	3.9 (7)	C15—O2—C17—O3	4.7 (8)
N1—C5—C6—O1	−4.1 (8)	C14—O3—C17—O2	−5.8 (8)
C4—C5—C6—O1	173.4 (5)		

Symmetry codes: (i) −*x*+1, −*y*+1, −*z*+1; (ii) −*x*, −*y*+1, −*z*+1.
